# Identifying transdiagnostic psychological processes that can improve early intervention in youth mental health

**DOI:** 10.1177/00048674241312803

**Published:** 2025-01-14

**Authors:** Tracey D Wade, Jamie-Lee Pennesi, Mia Pellizzer

**Affiliations:** Discipline of Psychology, Flinders University Institute of Mental Health and Wellbeing, Adelaide, SA, Australia

**Keywords:** Transdiagnostic, psychological processes, early intervention, youth, anxiety, depression, eating disorders

## Abstract

The aim of this viewpoint paper is to consider different psychological transdiagnostic processes that can inform the development of effective early intervention approaches in youth mental health before threshold diagnosis is attained. A transdiagnostic process is defined as a mechanism which is present across different disorders and is either a risk or a maintaining factor for the disorder. We consulted the literature with respect to processes across depression, anxiety and eating disorders. We suggest 38 unique transdiagnostic psychological processes. Each were defined to make them suitable for stakeholder consultation (e.g. people with lived experience) in developing transdiagnostic processes (targets) for youth early interventions. We recommend that the definitions of these processes are further developed in consultation with stakeholders, and that systematic reviews are conducted to further identify psychological processes that can inform essential ingredients of interventions that can then be tested for clinical impact in early intervention with youth.

## Introduction

It has been documented across numerous reviews that the mental health of youth (adolescents and emerging adult up to 25 years) has been declining over the last two decades. The Lancet Psychiatry Commission on youth mental health (McGorry et al., 2024) suggests a variety of influences could be responsible for this trend, including the rise of neoliberalism, the smartphone and unregulated social media, and greater pressure to achieve academically. A major surge in this trend was driven by the COVID-19 pandemic. For example, in Israel, significant increases in mental health diagnoses between 2017 and 2021 were documented in adolescents (Bilu et al., 2023). This increase was highest in eating disorders (50%), followed by depression (36%) and anxiety (31%), while psychiatric drug dispensation increased significantly by 28% for antipsychotics and 25% for antidepressants. These increases were most prominent among youth who were female, without a prior psychiatric history, of medium-high socioeconomic status and aged 14 to 15 (Bilu et al., 2023). In Australia, nearly 40% of youth aged 16–24 experience a mental health disorder, with more than one in four females engaging in self-harm in their lifetime (Australian Bureau of Statistics, 2023).

It is predicted that sustained effects of the pandemic will continue to be experienced by youth (Ipsos, 2024) due to persisting scars, including higher cost of living, heightened perceived vulnerability from the risk of another pandemic, erosion of social relationships, interrupted developmental milestones and impaired prospects related to interrupted academic progress (Ipsos, 2024; McGorry et al., 2024). The impact of climate change and regional conflicts adds to the perception that the future is bleak. In this context, the Australian Psychological Society (2024) has called for urgent investment in early intervention in youth mental health.

## Applying a transdiagnostic approach to early intervention

In our work, we are developing brief digital interventions for youth before a diagnosable disorder emerges. An international panel (Scott et al., 2024) has defined six stages in a transdiagnostic staging framework, three of which occur before a full-threshold disorder emerges. This includes no current symptoms but increased risk of a disorder (Stage 0), mild or non-specific symptoms (Stage 1a) and moderate but sub-threshold symptoms (Stage 1b). Given the high level of comorbidity across depression, anxiety disorders and eating disorders (Udo and Grilo, 2019), our intent is to build interventions that are limited, in the first instance, to target the broad symptomatology that may develop into one or more of these three disorders.

To achieve this aim, we have adopted a transdiagnostic approach that removes the distinctions between proposed psychiatric taxa at the level of classification and suggests alternative conceptualisations relating to processes implicated in mental health (Dalgleish et al., 2020). A process is a mechanism that is present across different disorders and is either a risk or a maintaining factor for the disorder, which will result in a symptom. The National Institute of Mental Health Research Domain Criteria (RDoC; Cuthbert, 2015) is one such example, limiting processes to dysfunctions in neural circuitry with a view to developing an alternative system of mental illness classification (Lilienfeld and Treadway, 2016). This transdiagnostic approach enables novel ways of thinking about onset, maintenance, clinical treatment and recovery from experiences of disabling mental distress. In the case of our research, we wish to link identified processes to elements or ingredients (defined as the essential conceptually well-defined aspects of an intervention that drive clinical impact; Sebastian et al., 2021; Wolpert et al., 2021), to examine the impact on a broad range of symptomatology. Understanding these elements allows researchers to move beyond knowing that a particular approach works to being able to identify its effective components to further refine and improve the treatment. An example can be found in the work of van den Heuvel et al. (2023) examining the most effective components of cognitive behaviour therapy (CBT) for depression in adolescents.

**Figure 1.**
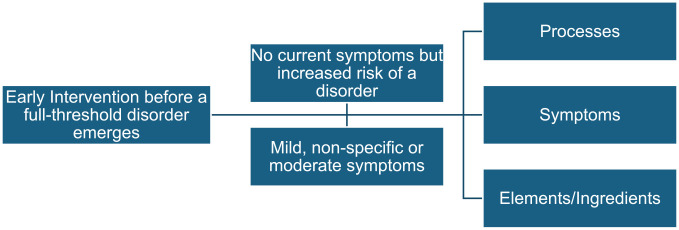
Components of early intervention.

As indicated above and shown in Figure 1, we suggest that the development of effective transdiagnostic early intervention approaches before a full-threshold disorder emerges requires consideration of three components to increase leverage for personalisation of interventions, regardless of the specific disorder that may eventually develop (Nye et al., 2023). These components include processes or mechanisms, elements or ingredients and symptoms. Sauer-Zavala et al. (2017) describe the way in which these three components interact. In a shared mechanisms treatment, resultant intervention components (elements) explicitly target common underlying mechanisms or processes that are relevant across a class of disorders where effectiveness is shown by a decrease in symptoms. Thus, the strategies included in a shared mechanisms approach are informed by theoretical models of psychopathology and are explicitly designed to target core features that occur across disorders. In their meta-analytic examination of interventions to prevent the onset of major depression, Buntrock et al. (2024) emphasise the importance of tailoring content to individuals’ specific challenges and experiences (i.e. processes) to further improve the effectiveness of preventive psychological interventions for subthreshold depressive symptoms. Our intent in this Viewpoint article is to elaborate further on the transdiagnostic processes relevant across depression, anxiety and eating disorders to better inform the development of effective transdiagnostic early intervention (Dalgleish et al., 2020; Skivington et al., 2021).

While transdiagnostic interventions have not to date produced more effective interventions than diagnostic specific interventions (Fusar-Poli et al., 2019), there is an ‘overwhelming weight of evidence from decades of such data-driven efforts . . . that mental health problems are best conceptualized along a series of continua rather than as discrete categories’ (Dalgleish et al., 2020: 182). For example, hierarchical dimensions predict significantly greater variance in eating disorder behaviours and future impairment than diagnosis (Forbush et al., 2024).

## Aims of this Viewpoint article

It has been suggested that a focus on transdiagnostic processes that are relevant to the development and maintenance of a range of diagnoses will lead to more robust treatment approaches that efficiently address symptoms of multiple disorders (Mansell et al., 2008; Sauer-Zavala et al., 2017). Despite this ambitious aim, researchers have struggled to leave disorder-specific confines when considering development of transdiagnostic approaches. For example, it is not unusual for transdiagnostic approaches to be applied explicitly only to anxiety and depression (Craske, 2012; Sebastian et al., 2021). In addition, the transdiagnostic CBT-enhanced model (CBT-E; Fairburn et al., 2003) was created to address only the full range of eating disorder diagnoses. In pursuit of developing brief digital interventions for youth before a diagnosable disorder emerges, we propose to take one step forward and identify psychological transdiagnostic processes across depression, anxiety disorders and eating disorders, to develop intervention elements that can impact a range of symptoms. It is also our aim to describe these processes in a way that can be understood by lay audiences in order to facilitate future co-design (involvement of relevant stakeholders), which is considered essential for the development of effective interventions (Skivington et al., 2021), consistent with the statement on consumer and community involvement in health and medical research co-authored by the National Health and Medical Research Council and the Consumers Health Forum of Australia.^1^

## Identifying psychological transdiagnostic processes

Early pioneering work (Harvey et al., 2004; Mansell et al., 2008) examined 14 psychological transdiagnostic processes, grouped in five categories, summarised in Table 1. A later review identified 16 transdiagnostic processes (Morris and Mansell, 2018), with new entries featuring perfectionism and intolerance of uncertainty.

**Table 1.** Transdiagnostic processes postulated by Harvey et al. (2004) as summarised in the study by Dalgleish et al. (2020).

Category	Specific processes
Attention	Selective attention (external), selective attention (internal), attentional avoidance
Memory	Explicit selective memory, recurrent memories, over general memories
Reasoning	Interpretational bias, expectancy bias, emotional reasoning
Thought	Recurrent negative thinking, positive and negative metacognitive beliefs, thought suppression
Behaviour	Avoidance, safety behaviours

After consideration of the extant reviews in this area, our recent review and meta-analysis of augmentations to eating disorder treatment (Pennesi et al., 2024), as well as a meta-analysis of transdiagnostic early interventions in youth (Radunz et al., manuscript submitted for publication), we adopted a quasi-quantitative approach to suggest 38 potential transdiagnostic processes described in Table 2 in such a way that makes them suitable for initial stakeholder consultation. Each is supported by some degree of evidence (see Supplementary Material) relating to status as a risk or maintenance factor of relevance across at least three different indicators of mental distress (i.e. anxiety, depression, eating disorders). Not included in the table are eight processes that were identified for only two of our three areas of symptomatology; difficulties with problem solving, explicit selective memory and thought suppression (evidence lacking for eating disorders) and heightened interoceptive awareness, low central coherence, low optimism, poor interoceptive awareness and unhelpful thinking habits (evidence lacking for anxiety disorders).

**Table 2.** Description of hypothesised transdiagnostic processes across anxiety disorders, depression and eating disorders.

Name	Description	Evidence of a link between the process and psychopathology
Psychophysiological		
Anxiety sensitivity	An intense fear of anxiety-related bodily sensations (such as increased heart rate), based on negative beliefs about the meaning and consequences of the sensations (e.g. mistakenly fearing one will die due to elevated heart rate)	Anxiety disorder (Olatunji and Wolitzky-Taylor, 2009) Depression (Olatunji and Wolitzky-Taylor, 2009) Eating disorder (Bazo Perez et al., 2023)
Poor nutrition	Poor nutrition unrelated to appearance concerns (e.g. due to food insecurity or from following a restrictive diet for a purpose unrelated to weight such as for health, ethical or medical reasons).	Anxiety disorder (Collins et al., 2022) Depression (Collins et al., 2022) Eating disorder (Hazzard et al., 2020)
Sleep problems	Sleep problems such as difficulty falling or staying asleep	Anxiety disorder (Scott et al., 2021) Depression (Scott et al., 2021) Eating disorder (Degasperi et al., 2024)
External exposures		
Appearance-focused physical activity	Participating in physical activities and sports that emphasise a certain body shape, weight, or appearance (e.g. classical ballet or body building)	Anxiety disorder (Cerea et al., 2018) – body builders Depression (Hallsworth et al., 2005) – body builders Eating disorder (Hallsworth et al., 2005; Prichard & Tiggemann, 2008)
Bullying	Receiving repeated intimidation, teasing, threats and physical aggression intended to cause physical or emotional distress	Anxiety disorder (Moore et al., 2017) Depression (Moore et al., 2017) Eating disorder (Fairweather-Schmidt & Wade, 2017)
Life transitions	Difficulty coping with developmental life transitions challenges and change (e.g. moving into adolescence or early adulthood)	Anxiety disorder (Lee and Gramotnev, 2007) Depression (Lee and Gramotnev, 2007) Eating disorder (Breton et al., 2022)
Negative social media use	Social media use focused on negative or distressing content (e.g. appearance focused content about how one should act and look, content related to self-harm and suicide)	Anxiety disorder (Sohn et al., 2019) Depression (Sohn et al., 2019) Eating disorder (de Valle et al., 2021)
Trauma	Exposure to traumatic life experiences that have resulted in shame or a sense of being deficient as a person (e.g. blaming oneself and feeling guilt and shame after being assaulted)	Anxiety disorder (Fernandes & Osório, 2015) Depression (Vibhakar et al., 2019) Eating disorder (Molendijk et al., 2017)
Social isolation	Difficulty getting the social support one feels is needed to manage the challenges of life	Anxiety disorder (Teo, Lerrigo et al., 2013) – social anxiety Depression (Teo et al., 2013) Eating disorder (Stern et al., 2023)
Cognitive		
Cognitive biases including selective attention to or interpretation of threat	The tendency to notice or pay attention to some things more than others (e.g. only noticing possible signs of danger and overlooking signs that the environment is safe) or interpreting things in negative and unhelpful ways (e.g. interpreting neutral facial expressions as negative, such as thinking others do not like oneself)	Anxiety disorder (Dudeney et al., 2015) – children Depression (Nieto et al., 2020) Eating disorder (Stott et al., 2021)
Difficulties with mindful observation	The ability to step back and observe emotion and situations without judgement and without reacting to them	Anxiety disorder (Enkema et al., 2020) Depression (Enkema et al., 2020) Eating disorder (Omiwole et al., 2019)
Harm avoidance	A tendency to anticipate and avoid possible threats and risks due to anxiety (e.g. fearing and avoiding conversations with people you do not know because of the fear you might be boring)	Anxiety disorder (Cervin et al., 2020) Depression (Cervin et al., 2020) Eating disorder (Atiye et al., 2015)
Impaired theory of mind	Difficulty understanding that others’ beliefs, desires, intentions, emotions and thoughts may be different from one’s own (e.g. assuming someone has the same knowledge as you about a specific subject)	Anxiety disorder (Baez et al., 2023) Depression (Nestor et al., 2022) Eating disorder (Preti et al., 2022) – anorexia nervosa
Low levels of psychological/cognitive flexibility	Having a hard time adjusting to changes or new situations (e.g. feeling uncomfortable taking a different route to work if there are road works)	Anxiety disorder (Zainal and Newman, 2018) Depression (Liu et al., 2021) Eating disorder (Keegan et al., 2021)
Overgeneral memory (overgeneral autobiographical memories)	Remembering broad, general details about past events and having difficulty remembering specific moments and details	Anxiety disorder (Ono et al., 2016)–PTSD Depression (Barry et al., 2021) Eating disorder (Barry et al., 2021)
Recurring (negative) memories (recurrent involuntary memories)	Memories that come back to your mind repeatedly, often without one wanting them to (e.g. remembering an argument with a loved one over and over)	Anxiety disorder (Yeung and Fernandes, 2021) Depression (Yeung and Fernandes, 2021) Eating disorder (Kadriu et al., 2022)
Repetitive negative thinking and rumination	Being stuck in a cycle of negative thoughts where one repeatedly thinks about the same thoughts over and over again	Anxiety disorder (Rickerby et al., 2024) Depression (Rickerby et al., 2024) Eating disorder (Rickerby et al., 2024)
Use of safety behaviours	Using strategies to avoid a feared consequence of anxiety to feel safer or more in control but they make anxiety worse over time (e.g. practising what to say in a conversation may temporarily decrease social anxiety, however, if this makes conversation flow difficult and awkward, this could increase the anxiety)	Anxiety disorder (Fledderus et al., 2010) Depression (Fledderus et al., 2010) Eating disorder (Rawal et al., 2010)
Other psychological		
Absence of a clear personal identity and sense of self	Not knowing who you are as a person and the thoughts, feelings, values and experiences that are important to you and shape your identity (e.g. trust, loyalty, compassion)	Anxiety disorder (Potterton et al., 2022) Depression (Potterton et al., 2022) Eating disorder (Potterton et al., 2022)
High need for control	An obsessional need to control environment (e.g. ordering things just so, needing a certain routine)	Anxiety disorder (Moulding et al., 2008) – OCD Depression (Myles et al., 2020) Eating disorder (Roberts et al., 2011) – anorexia nervosa
Impulsivity and risk taking	Acting quickly without thinking through the consequences (e.g. alcohol and drug use, having unprotected sex with a stranger)	Anxiety disorder (Berg et al., 2015) Depression (Berg et al., 2015) Eating disorder (Berg et al., 2015)
Intolerance of uncertainty	Finding uncertainty (not being sure about what will happen next) stressful and uncomfortable. One might try to avoid uncertainty or find it difficult to manage when it does happen	Anxiety disorder (McEvoy et al., 2019) Depression (McEvoy et al., 2019) Eating disorder (McEvoy et al., 2019)
Loneliness	Feeling isolated and disconnected from others	Anxiety disorder (Loades et al., 2020) Depression (Loades et al., 2020) Eating disorder (Hanna et al., 2023)
Low positive affectivity	A tendency to experience positive emotions, such as enthusiasm and joy, less than other people	Anxiety disorder (Khazanov and Ruscio, 2016) Depression (Khazanov and Ruscio, 2016) Eating disorder (Haynos et al., 2021)
Low self-compassion	Difficulty with the ability to show compassion and kindness towards oneself	Anxiety disorder (MacBeth and Gumley, 2012) Depression (MacBeth and Gumley, 2012) Eating disorder (Turk and Waller, 2020)
Low self-efficacy and empowerment	Having a low level of the belief that one has the ability and power to complete a task or achieve a goal	Anxiety disorder (Tahmassian and Moghadan, 2016) Depression (Santos et al., 2013) Eating disorder (Bardone-Cone et al., 2006)
Low self-worth and self-acceptance	Not feeling good enough, worthy of love from others and difficulty accepting oneself	Anxiety disorder (Henriksen et al., 2022) Depression (Henriksen et al., 2022) Eating disorder (Krauss et al., 2023)
Negative affectivity	A tendency to experience negative emotions, such as anger, guilt and fear, more than other people	Anxiety disorder (Gulley et al., 2016) Depression (Gulley et al., 2016) Eating disorder (Dufresne et al., 2020)
Negative body image	Being dissatisfied and unhappy with, and having negative thoughts and feelings about, one’s body, weight or shape	Anxiety disorder (Barnes et al., 2020)–Men Depression (Barnes et al., 2020) Eating disorder (Stice et al., 2017)
Negative emotion avoidance	Avoidance of expression of negative emotion by self and others (such as avoiding showing anger or avoiding others who are expressing anger)	Anxiety disorder (Akbari et al., 2022) Depression (Akbari et al., 2022) Eating disorder (Leppanen et al., 2022)
Perfectionism – Fear of mistakes	Fear of mistakes (e.g. selective attention to mistakes and imperfection [noticing them more than things they do fine or well] resulting in self-criticism and beliefs that others think less of them as a person)	Anxiety disorder (Limburg et al., 2017) Depression (Limburg et al., 2017) Eating disorder (Limburg et al., 2017)
Perfectionism – High standards	Persistent, excessively high standards (e.g. I must have the best grade in all my subjects to feel good about myself)	Anxiety disorder (Limburg et al., 2017) Depression (Limburg et al., 2017) Eating disorder (Limburg et al., 2017)
Poor distress tolerance skills and emotion regulation problems	Finding it difficult to identify and manage one’s emotion	Anxiety disorder (Schäfer et al., 2016) Depression (Schäfer et al., 2016) Eating disorder (Leppanen et al., 2022)
Poor emotion recognition in others (theory of mind)	Finding it difficult to identify and understand what emotion someone else is experiencing	Anxiety disorder (Baez et al., 2023) Depression (Dalili et al., 2015) Disordered eating eating disorder (Leppanen et al., 2022)
Poor social skills	A lack of the skills we use to communicate with others in daily life, including verbal (what we say and how) and non-verbal communication (such as our body language, gestures, and facial expressions).	Anxiety Disorder (Segrin, 2019) – indirect effects via loneliness/stress Depression (Segrin, 2019) – as above Eating Disorder (Uzunian & Vitalle, 2015)
Self-critical	A tendency to criticise and judge oneself harshly. It is like having an inner voice that constantly points out what you are doing wrong	Anxiety disorder (Dunkley et al., 2020) Depression (Dunkley et al., 2020) Eating disorder (Paranjothy & Wade, 2024)
Self-silencing and submissiveness	When a person suppresses their own thoughts, feelings or needs to please others, avoid conflict or because they do not feel a legitimate right to express themselves. Also, a tendency to do what others want or ask without questioning them	Anxiety disorder (Kosmicki, 2017) Depression (Emran et al., 2020) Eating disorder (Emran et al., 2020)
Self-worth	Basing self-worth on just one or two aspects of oneself, including achievement, being perfect, appearance or control over weight/eating	Anxiety disorder (Bardone-Cone et al., 2017) Depression (Lakey et al., 2014) Eating disorder (Bardone-Cone et al., 2017)

## Future directions

While this work represents one step forward in the identification of psychological transdiagnostic processes across depression, anxiety disorders and eating disorders, there is much further work required to see these translated to effective early intervention. First, we have submitted the list of transdiagnostic processes for consultation to stakeholders (i.e. people with lived experience, carers and significant others, clinicians and researchers) to further refine the descriptions, indicate omissions and identify consensus about the transdiagnostic processes that are most important to tackle in youth early intervention. Second, to further refine this list of transdiagnostic processes – either removing or adding processes or collapsing some processes – we have initiated a systematic review process. We will search for meta-analyses or systematic reviews related to mechanisms across the different psychopathologies. These two complementary processes can then inform development of elements to target in early interventions that can be tested in randomised controlled trials. To date, it is clear, that the evidence provided to support status as a transdiagnostic process is uneven and indicates gaps that need to be addressed in future research.

## Supplemental Material

sj-docx-1-anp-10.1177_00048674241312803 – Supplemental material for Identifying transdiagnostic psychological processes that can improve early intervention in youth mental health Supplemental material, sj-docx-1-anp-10.1177_00048674241312803 for Identifying transdiagnostic psychological processes that can improve early intervention in youth mental health by Tracey D Wade, Jamie-Lee Pennesi and Mia Pellizzer in Australian & New Zealand Journal of Psychiatry

## Notes

1.
https://www.nhmrc.gov.au/about-us/publications/statement-consumer-and-community-involvement-health-and-medical-research
