# Supplementary material for: Identifying transdiagnostic psychological processes that can improve early intervention in youth mental health
Source: Aust N Z J Psychiatry. 2025 Jan 14;59(4):307–14. doi: 10.1177/00048674241312803 (PMC11924278; doi:10.1177/00048674241312803)
Supplement: sj-docx-1-anp-10.1177_00048674241312803 – Supplemental material for Identifying transdiagnostic psychological processes that can improve early intervention in youth mental health [file sj-docx-1-anp-10.1177_00048674241312803.docx]

Word count : 1443/3000

Tables: 2

Abstract: 193/200

22^nd^ November 2024

**Identifying Transdiagnostic Processes that can improve Early Intervention in Youth Mental Health**

Tracey D Wade, PhD ORCID: 0000-0003-4402-770X

Jamie-Lee Pennesi, PhD ORCID: 0000-0003-1461-7303

Mia Pellizzer, PhD ORCID: [0000-0002-9917-2069](https://orcid.org/0000-0002-9917-2069)

Flinders University Institute of Mental Health and Wellbeing, South Australia, Australia

**For Submission to**: Australian and New Zealand Journal of Psychiatry (Viewpoint article)

**Supplementary Material: References in Table 2**

Akbari M, Seydavi M, Hosseini ZS et al (2022) Experiential avoidance in depression, anxiety, obsessive-compulsive related, and posttraumatic stress disorders: A comprehensive systematic review and meta-analysis. *Journal of Contextual Behavioral Science* 24: 65-78.

Atiye M, Miettunen J, Raevuori-Helkamaa A (2015) A meta-analysis of temperament in eating disorders. *European Eating Disorders Review* 23: 89–99.

Baez S, Tangarife MA, Davila-Mejia G, et al (2023) Performance in emotion recognition and theory of mind tasks in social anxiety and generalized anxiety disorders: a systematic review and meta-analysis. *Frontiers in Psychiatry* 14: 1192683.

Bardone-Cone AM, Abramson LY, Vohs KD et al (2006) Predicting bulimic symptoms: An interactive model of self-efficacy, perfectionism, and perceived weight status. *Behaviour Research and Therapy* 44(1): 27–42.

Bardone-Cone AM, Lin SL, Butler RM (2017) Perfectionism and contingent self-worth in relation to disordered eating and anxiety. *Behavior Therapy* 48(3): 380-390.

Barnes M, Abhyankar P, Dimova E, et al (2020) Associations between body dissatisfaction and self-reported anxiety and depression in otherwise healthy men: A systematic review and meta-analysis. *PloS One* 15(2): e0229268.

Barry TJ, Hallford DJ, Takano K (2021) Autobiographical memory impairments as a transdiagnostic feature of mental illness: A meta-analytic review of investigations into autobiographical memory specificity and overgenerality among people with psychiatric diagnoses. *Psychological Bulletin* 147(10): 10541074.

Bazo Perez M, Hayes TB, Frazier LD (2023) Beyond generalized anxiety: the association of anxiety sensitivity with disordered eating. *Journal of Eating Disorders* 11(1): 173.

Berg JM, Latzman RD, Bliwise NG et al (2015) Parsing the heterogeneity of impulsivity: A meta-analytic review of the behavioral implications of the UPPS for psychopathology. *Psychological Assessment* 27(4): 1129.

Breton É, Dufour R, Côté SM, et al (2022) Developmental trajectories of eating disorder symptoms: A longitudinal study from early adolescence to young adulthood. Journal of Eating Disorders 10(1): 84.

Cerea S, Bottesi G, Pacelli QF et al (2018) Muscle Dysmorphia and its Associated Psychological Features in Three Groups of Recreational Athletes. *Science Reports* 8: 8877.

Cervin M, Perrin S, Olsson E, et al (2020) Incompleteness, harm avoidance, and disgust: A comparison of youth with OCD, anxiety disorders, and no psychiatric disorder. *Journal of Anxiety Disorders* 69: 102175.

Collins S, Dash S, Allender S, et al (2022) Diet and Mental Health During Emerging Adulthood: A Systematic Review. *Emerging Adulthood* 10(3): 645-659.

Dalili MN, Penton-Voak IS, Harmer CJ, et al (2015) Meta-analysis of emotion recognition deficits in major depressive disorder. *Psychological Medicine* 45(6): 1135-1144.

de Valle MK, Gallego-García M, Williamson P, et al (2021) Social media, body image, and the question of causation: Meta-analyses of experimental and longitudinal evidence. *Body Image* 39: 276–292.

Degasperi G, Meneo D, Curati S, et al (2024) Sleep quality in eating disorders: A systematic review and meta-analysis. Sleep Medicine Reviews 18;77: 101969.

Dudeney, J, Sharpe L, Hunt C (2015) Attentional bias towards threatening stimuli in children with anxiety: A meta-analysis. *Clinical Psychology Review* 40: 66-75.

Dufresne L, Bussières EL, Bédard A, et al (2020) Personality traits in adolescents with eating disorder: A meta‐analytic review. *International Journal of Eating Disorders* 53(2): 157-173.

Dunkley DM, Starrs CJ, Gouveia L et al (2020) Self-critical perfectionism and lower daily perceived control predict depressive and anxious symptoms over four years. *Journal of Counseling Psychology* *67*(6): 736–746.

Eggart M, Lange A, Binser MJ, et al (2019) Major depressive disorder is associated with impaired interoceptive accuracy: A systematic review. *Brain Sciences* 9(6): 131.

Emran A, Iqbal N, and Dar IA (2020) ‘Silencing the self’ and women’s mental health problems: A narrative review. *Asian Journal of Psychiatry* 53: 102197.

Enkema MC, McClain L, Bird ER, et al (2020) Associations between mindfulness and mental health outcomes: A systematic review of ecological momentary assessment research. *Mindfulness* 11(11): 2455–2469.

Everaert J, Vrijsen JN, Martin-Willett R, et al (2022) A meta-analytic review of the relationship between explicit memory bias and depression: Depression features an explicit memory bias that persists beyond a depressive episode. *Psychological Bulletin* 148(5-6): 435-463.

Fairweather-Schmidt AK, Wade TD (2017) Weight-related peer-teasing moderates genetic and environmental risk and disordered eating: twin study. *The British Journal of Psychiatry* 210: 350–355.

Fernandes V, Osório FL (2015) Are there associations between early emotional trauma and anxiety disorders? Evidence from a systematic literature review and meta-analysis. *European Psychiatry* 30(6): 756-764.

Fledderus M, Bohlmeijer ET, Pieterse ME. (2010) Does experiential avoidance mediate the effects of maladaptive coping styles on psychopathology and mental health? Behavior Modification 34(6): 503-19.

Galente J, Friedrich C, Dalgleish T, Jones PB, White IR (2023). Individual participant data systematic review and meta-analysis of randomised controlled trials assessing adult mindfulness-based programmes for mental health promotion in non-clinical settings. *Nature Mental Health* 10: 462-476.

Grogan K, MacGarry D, Bramham J, et al (2020) Family-related non-abuse adverse life experiences occurring for adults diagnosed with eating disorders: a systematic review. *Journal of Eating Disorders* 8: 1-20.

Gulley LD, Hankin BL, Young JF (2016) Risk for depression and anxiety in youth: The interaction between negative affectivity, effortful control, and stressors. *Journal of Abnormal Child Psychology* 44: 207-218.

Hallsworth L, Wade TD, Tiggemann M (2005) Individual differences in male body-image: an examination of self-objectification in recreational body builders. *British Journal of Health Psychology* 10: 453–465.

Hanna K, Cross J, Nicholls A, et al (2023) The association between loneliness or social isolation and food and eating behaviours: A scoping review. *Appetite* 191: 107051.

Haynos AF, Anderson LM, Askew AJ et al (2021) Adapting a neuroscience-informed intervention to alter reward mechanisms of anorexia nervosa: a novel direction for future research. Journal of Eating Disorders 9: 63.

Hazzard VM, Loth KA, Hooper, L et al (2020) Food Insecurity and Eating Disorders: a Review of Emerging Evidence. *Current Psychiatry Reports* 22: 74.

Heeke C Kampisiou C, Niemeyer H et al (2017) A systematic review and meta-analysis of correlates of prolonged grief disorder in adults exposed to violent loss. *European Journal of Psychotraumatology* 8: 1583524.

Henriksen IO, Ranøyen I, Indredavik MS, et al (2017) The role of self-esteem in the development of psychiatric problems: a three-year prospective study in a clinical sample of adolescents. *Child and Adolescent Psychiatry and Mental Health* 11:68.

Hermesdorf M, Berger K, Baune BT et al (2016) Pain Sensitivity in Patients With Major Depression: Differential Effect of Pain Sensitivity Measures, Somatic Cofactors, and Disease Characteristics. *Journal of Pain* 17(5): 606-16.

Herrera S, Montorio I, Cabrera I, et al (2017). Memory bias for threatening information related to anxiety: An updated meta-analytic review. *Journal of Cognitive Psychology* 29(7): 832-854.

Jenkinson PM, Taylor L, Laws KR (2018) Self-reported interoceptive deficits in eating disorders: A meta-analysis of studies using the eating disorder inventory. *Journal of Psychosomatic Research* 110: 38-45.

Kadriu F, Claes L, Witteman C, et al (2022). Intrusive images, autobiographical memories, and core beliefs of patients with an eating disorder. *Applied Cognitive Psychology* 36(4): 842–851.

Keegan E, Tchanturia K, Wade TD (2021) Central coherence and set shifting between non underweight eating disorders and anorexia nervosa: A systematic review and meta-analysis.  *International Journal of Eating Disorders* 54(3): 229–243.

Khazanov GK, Ruscio AM (2016) Is low positive emotionality a specific risk factor for depression? A meta-analysis of longitudinal studies. *Psychological Bulletin* 142(9), 991.

Komischke-Konnerup KB, Zachariae R, Johannsen M, et al (2021) Co-occurrence of prolonged grief symptoms and symptoms of depression, anxiety, and posttraumatic stress in bereaved adults: A systematic review and meta-analysis. *Journal of Affective Disorders Reports* 4: 100140.

Kosmicki MT (2017). Marianismo Identity, Self-Silencing, Depression and Anxiety in Women from Santa María de Dota, Costa Rica. *UNED Research Journal* 9:202-208.

Krauss S, Dapp LC, Orth U (2023) The Link Between Low Self-Esteem and Eating Disorders: A Meta-Analysis of Longitudinal Studies. *Clinical Psychological Science* 11(6): 1141-1158.

Lakey CE, Hirsch JK, Nelson LA et al (2014). Effects of contingent self-esteem on depressive symptoms and suicidal behavior. *Death studies*, *38*(9), 563-570.

Lee, C., & Gramotnev, H. (2007). Life transitions and mental health in a national cohort of young Australian women. *Developmental Psychology* 43(4): 877–888.

Leppanen J, Brown D, McLinden H, et al (2022) The role of emotion regulation in eating disorders: a network meta-analysis approach. *Frontiers in Psychiatry* 13: 793094.

Limburg K, Watson HJ, Hagger MS et al (2017) The relationship between perfectionism and psychopathology: A meta-analysis. *Journal of Clinical Psychology* 73: 1301-1326.

Liu H, Funkhouser CJ, Langenecker SA et al (2021) Set Shifting and Inhibition Deficits as Potential Endophenotypes for Depression. *Psychiatry Research* 300: 113931.

Loades ME, Chatburn E, Higson-Sweeney N, et al (2020) Rapid Systematic Review: The Impact of Social Isolation and Loneliness on the Mental Health of Children and Adolescents in the Context of COVID-19. *Journal of the American Academy of Child and Adolescent Psychiatry* 59(11): 1218–1239.e3.

MacBeth A, Gumley A (2012) Exploring compassion: a meta-analysis of the association between self-compassion and psychopathology. *Clinical Psychology Review* 32(6): 545–552.

Mamat Z, Anderson MC (2023) Improving mental health by training the suppression of unwanted thoughts. *Science Advances* 22;9(38): eadh5292.

McDermott LM, Ebmeier KP (2009) A meta-analysis of depression severity and cognitive function. *Journal of Affective Disorders* 119: 1–8.

McEvoy PM, Hyett MP, Shihata S, et al (2019) The impact of methodological and measurement factors on transdiagnostic associations with intolerance of uncertainty: A meta-analysis. *Clinical Psychology Review* 73: 101778.

Michelson D, Hodgson E, Bernstein A, et al (2022) Problem Solving as an Active Ingredient in Indicated Prevention and Treatment of Youth Depression and Anxiety: An Integrative Review. *The Journal of Adolescent Health* 71(4): 390–405.

Molendijk ML, Hoek HW, Brewerton TD et al (2017) Childhood maltreatment and eating disorder pathology: a systematic review and dose-response meta-analysis. *Psychological Medicine* 47: 1402-16.

Moore SE, Norman RE, Suetani S, et al (2017) Consequences of bullying victimization in childhood and adolescence: A systematic review and meta-analysis. *World Journal of Psychiatry*,7(1): 60–76.

Moulding R, Doron G, Kyrios M et al (2008) Desire for control, sense of control and obsessive-compulsive checking: An extension to clinical samples. *Journal of Anxiety Disorders* 22(8): 1472-1479.

Myles LAM, Connolly J and Stanulewicz N (2020). The mediating role of perceived control and desire for control in the relationship between personality and depressive symptomology. *Mediterranean Journal of Clinical Psychology* 8(3): 1-24.

Nestor BA, Sutherland S, Garber J (2022) Theory of mind performance in depression: A meta-analysis. *Journal of Affective Disorders* 303: 233–244.

Nieto I, Robles E, Vazquez C (2020) Self-reported cognitive biases in depression: A meta-analysis. *Clinical Psychology Review* 82: 101934.

Olatunji BO, Wolitzky-Taylor KB (2009) Anxiety sensitivity and the anxiety disorders: a meta-analytic review and synthesis. *Psychological Bulletin* 135(6): 974.

Omiwole M, Richardson C, Huniewicz P, et al (2019) Review of Mindfulness-Related Interventions to Modify Eating Behaviors in Adolescents. *Nutrients* 11(12): 2917.

Ono M, Devilly GJ, Shum DHK (2016) A meta-analytic review of overgeneral memory: The role of trauma history, mood, and the presence of posttraumatic stress disorder. *Psychological Trauma: Theory, Research, Practice, and Policy* 8(2): 157–164

Paranjothy SM, Wade TD (2024) A meta-analysis of disordered eating and its association with self-criticism and self-compassion. *International Journal of Eating Disorders* 57(3): 473–536.

Phillips L, Tiggemann M, Wade T. (1997) Comparison of cognitive style in bulimia nervosa and depression. *Behaviour Research and Therapy* 35(10): 939-48.

Potterton R., Austin A, Robinson L et al (2022) Identity Development and Social-Emotional Disorders During Adolescence and Emerging Adulthood: A Systematic Review and Meta-Analysis. *Journal of Youth and Adolescence* 51(1): 16–29.

Preti A, Siddi S, Marzola E, et al (2022) Affective cognition in eating disorders: a systematic review and meta-analysis of the performance on the "Reading the Mind in the Eyes" Test. *Eating and Weight Disorders* 27(7): 2291–2307.

Prichard I, Tiggemann M (2008) Relations among exercise type, self-objectification, and body image in the fitness centre environment: The role of reasons for exercise. *Psychology of Sport and Exercise* 9(6): 855-866.

Raes F, Griffith JW, Craeynest M, et al (2023) Overgeneralization as a predictor of the course of depression over time: The role of negative overgeneralization to the self, negative overgeneralization across situations, and overgeneral autobiographical memory. *Cognitive Therapy and Research* 47(4): 598–613.

Rawal A, Park RJ, Williams JM. (2010) Rumination, experiential avoidance, and dysfunctional thinking in eating disorders. *Behaviour Research and Therapy* 48(9): 851-9.

Rickerby N, Krug I, Fuller-Tyszkiewicz M, et al (2022) Rumination across depression, anxiety, and eating disorders in adults: A meta-analytic review. *Clinical Psychology: Science and Practice* 31(2): 251–268

Robert M, Buscail C, Allès B, et al (2020) Dispositional optimism is associated with weight status, eating behavior, and eating disorders in a general population-based study. *International Journal of Eating Disorders* 53(10): 1696-1708.

Roberts M, Lavender A and Tchanturia, K (2011) Measuring self-report obsessionality in anorexia nervosa: Maudsley Obsessive-Compulsive Inventory (MOCI) or obsessive-compulsive inventory-revised (OCI-R)? *European Eating Disorders Review* 19(6): 501–508.

Santos HP, Kossakowski JJ, Schwartz TA et al (2018) Longitudinal network structure of depression symptoms and selfefficacy in low-income mothers. *PLoS ONE* 13(1): e0191675.

Schäfer JÖ, Naumann E, Holmes EA, et al (2017) Emotion regulation strategies in depressive and anxiety symptoms in youth: A meta-analytic review. *Journal of Youth and Adolescence* 46: 261-276.

Scott AJ, Webb TL, Martyn-St James M, et al (2021) Improving sleep quality leads to better mental health: A meta-analysis of randomised controlled trials. *Sleep Medicine Review* 60: 101556.

Segrin C (2019) Indirect Effects of Social Skills on Health Through Stress and Loneliness. *Health Communication* 34(1): 118-124.

Sohn SY, Rees P, Wildridge B, et al (2019) Prevalence of problematic smartphone usage and associated mental health outcomes amongst children and young people: a systematic review, meta-analysis and GRADE of the evidence. *BMC Psychiatry* 19(1): 356.

Stern M, Rubino L, Desjardins C et al (2023) Prospective reciprocal relations between social support and eating disorder symptoms. *Journal of Psychopathology and Clinical Science* 132(8): 1043–1050.

Stice E, Gau JM, Rohde P, et al (2017) Risk factors that predict future onset of each DSM-5 eating disorder: Predictive specificity in high-risk adolescent females. *Journal of Abnormal Psychology* 126: 38-51.

Stott N, Fox JR, Williams MO (2021) Attentional bias in eating disorders: A meta‐review. *International Journal of Eating Disorders* 54(8): 1377-1399.

Tahmassian K, Moghadam NJ (2011) Relationship between self-efficacy and symptoms of anxiety, depression, worry and social avoidance in a normal sample of students. *Iranian Journal of Psychiatry and Behavioral Sciences* 5(2): 91–98.

Teo AR, Choi H, Valenstein M (2013) Social relationships and depression: ten-year follow-up from a nationally representative study. *PLoS One* 30;8(4) :e62396.

Teo AR, Lerrigo R, Rogers MA. (2013) The role of social isolation in social anxiety disorder: a systematic review and meta-analysis. Journal of Anxiety Disorders 27(4): 353-64.

Turk F, Waller G (2020) Is self-compassion relevant to the pathology and treatment of eating and body image concerns? A systematic review and meta-analysis. *Clinical Psychology Review* 79*:* 101856.

Uribe FAR, de Oliveira SB, Junior AG et al (2021) Association between the dispositional optimism and depression in young people: a systematic review and meta-analysis. *Psychology: Research and Review* 34: 37.

Uzunian LG, Vitalle MS (2015) Social skills: a factor of protection against eating disorders in adolescents. *Ciência & Saúde Coletiva* 20(11): 3495-508.

Vibhakar V, Alle LR, Gee B et al (2019) A systematic review and meta-analysis on the prevalence of depression in children and adolescents after exposure to trauma. *Journal of Affective Disorders* 255: 77-89.

Yamamotova A, Bulant J Bocek V et al (2017) Dissatisfaction with own body makes patients with eating disorders more sensitive to pain. *Journal of Pain Research* 10: 1667-1675,

Yeung R, Fernandes M (2021) Recurrent involuntary memories are modulated by age and linked to mental health. *Psychology & Aging* 36, 883-890.

Zainal NH, Newman MG (2018) Executive function and other cognitive deficits are distal risk factors of generalized anxiety disorder 9 years later. *Psychological Medicine* 48(12): 2045–2053.
